# Remyelination in multiple sclerosis from the miRNA perspective

**DOI:** 10.3389/fnmol.2023.1199313

**Published:** 2023-06-01

**Authors:** Karina Maciak, Angela Dziedzic, Joanna Saluk

**Affiliations:** Department of General Biochemistry, Institute of Biochemistry, Faculty of Biology and Environmental Protection, University of Lodz, Lodz, Poland

**Keywords:** remyelination, miRNA, microRNA, multiple sclerosis, oligodendrocyte, demyelination, myelin, oligodendrocyte precursor cells

## Abstract

Remyelination relies on the repair of damaged myelin sheaths, involving microglia cells, oligodendrocyte precursor cells (OPCs), and mature oligodendrocytes. This process drives the pathophysiology of autoimmune chronic disease of the central nervous system (CNS), multiple sclerosis (MS), leading to nerve cell damage and progressive neurodegeneration. Stimulating the reconstruction of damaged myelin sheaths is one of the goals in terms of delaying the progression of MS symptoms and preventing neuronal damage. Short, noncoding RNA molecules, microRNAs (miRNAs), responsible for regulating gene expression, are believed to play a crucial role in the remyelination process. For example, studies showed that miR-223 promotes efficient activation and phagocytosis of myelin debris by microglia, which is necessary for the initiation of remyelination. Meanwhile, miR-124 promotes the return of activated microglia to the quiescent state, while miR-204 and miR-219 promote the differentiation of mature oligodendrocytes. Furthermore, miR-138, miR-145, and miR-338 have been shown to be involved in the synthesis and assembly of myelin proteins. Various delivery systems, including extracellular vesicles, hold promise as an efficient and non-invasive way for providing miRNAs to stimulate remyelination. This article summarizes the biology of remyelination as well as current challenges and strategies for miRNA molecules in potential diagnostic and therapeutic applications.

## 1. Introduction

Multiple sclerosis (MS) is a chronic autoimmune disease that leads to the deterioration of the myelin sheath in the brain, spinal cord, and optic nerves. It is a relatively common condition, with an estimated 2.8 million people worldwide living with the disease (“Atlas of MS 2020 – Epidemiology Report, 2020”). It represents a significant burden on healthcare systems, both in terms of the cost of treatment and the need for ongoing monitoring and care (Filippi et al., 2018).

There are four main subtypes of MS, each with unique features and patterns of disease progression. Relapsing–remitting (RR) MS is the most common form of the disease, characterized by relapses of neurological symptoms, followed by periods of remission, during which signs of the disease improve or disappear. Secondary-progressive (SP) MS follows the RRMS and is characterized by a steady progression of disability, with or without relapses and remissions. Primary-progressive (PP) MS is characterized by a gradual onset of symptoms that worsen over time, with little to no remission. Progressive-relapsing (PR) MS is a rare subtype, characterized by a steady progression of disability, with occasional relapses and remissions (Filippi et al., 2018).

Demyelination is a key feature of MS and is believed to play a central role in the pathogenesis of the disease (Lassmann, 2018). Activation of immune cells, such as T and B lymphocytes, defective functioning of regulatory cells, release of pro-inflammatory cytokines, including interferon (IFN)-γ and interleukin (IL)-17A, and neuroinflammation are key steps in the development of autoimmune demyelination in MS (Dendrou et al., 2015). Autoreactive T cells infiltrate the CNS and secrete cytokines that trigger macrophages, leading to the creation of demyelinating lesions in the white matter. As a result of the activation of T cells and the release of lymphokines, B cells are induced to transform into plasma cells, which generate autoantibodies that cause neurodegeneration by degrading the myelin sheath surrounding nerve fibers (Engelhardt and Ransohoff, 2005). The effect of immune cells-derived cytokines on glia and neurons causes damage to myelin membranes and a reduction in their integrity (Schmitz and Chew, 2008). Another important molecular aspect of demyelination in MS is the role of antigen-presenting cells (APCs) in the autoimmune process. APCs present myelin antigens on their surface, leading to the activation and proliferation of myelin-targeted autoreactive T cells and subsequent destruction of myelin membranes (van Langelaar et al., 2020). At first glance, demyelination in various CNS pathologies is quite similar; however, the stress to which brain cells are subjected during these pathologies is somewhat different. In contrast to MS, ischemic stroke (IS) is characterized by reduced cerebral blood flow, which limits the availability of glucose and oxygen (hypoxia), especially in neurons leading to immediate cell death (Qin et al., 2022), while trauma injury (TI) refers to severe sudden CNS damage, which requires instant medical attention (Ray et al., 2002). Contrary to chronic, multifocal demyelination of the CNS (brain and spinal cord) with clinical and/or radiological evidence of ‘dissemination in space’ (DIS, demyelination lesions at more than one place in the nervous system) and ‘dissemination in time’ (DIT, demyelination lesions have occurred more than once) in MS (Lopaisankrit and Thammaroj, 2023), major changes after IS and TI occur mainly in the brain microcirculation. With ongoing hypoxia/ischemia and circulating cell retention, there is a potential for vascular edema, preventing rapid recovery of normal blood flow after fluid resuscitation, which may result in immediate cell death. In both, disruption of energy leads to mitochondrial dysfunction and oxidative stress-induced injury, triggered by excessive production of reactive oxygen species (ROS; Hiebert et al., 2015; Jia et al., 2021). Simultaneously, energy deficiency contributes to an ionic imbalance that affects Na^+^, K^+^, and Ca^2+^ levels, causing the brain cell depolarization (mainly disturbances in macrophages pro-inflammatory and anti-inflammatory phenotype ratio) and inducing massive glutamate release, which activates N-methyl-D-aspartate receptors, inducing toxicity, severe injury, and finally CNS cell death (Ladak et al., 2019; Qin et al., 2022). According to our assumption, the key element that differentiates MS from other CNS pathologies is the possibility of remyelination. The process of demyelination in MS is mostly gradual, while in the case of IS or TI there is an immediate death of brain cells, with no chance of starting their repair mechanisms. Demyelination is a progressive process that causes permanent nerve damage and neurological loss. However, MS is characterized by the ability to remyelinate, i.e., repairing the myelin sheath surrounding the axons, which can be a natural process or the result of therapy (Chari, 2007).

Remyelination is the process by which CNS glial cells rebuild damaged myelin sheaths. Various cell types are involved in the remyelination process, including oligodendrocytes, oligodendrocyte precursor cells (OPCs), microglia, and astrocytes. Oligodendrocytes are the main myelin-producing cells in the CNS and are responsible for rebuilding myelin sheaths after damage. OPCs are stem cells that can differentiate into oligodendrocytes and play a role in remyelination. Microglia and astrocytes are crucial in the repair process, as they are involved in removing damaged cells and substances from the site of damage, as well as in the production of growth factors and cytokines (Uyeda and Muramatsu, 2020). A new myelin sheath can be formed by two different processes: remyelination or myelinogenesis. Remyelination is an intrinsic process of the repair of myelin within a partially damaged oligodendrocyte through cellular repair aided by cellular and non-cellular pro-repair extrinsic factors. Myelinogenesis relies on a replacement of a completely damaged oligodendrocyte by a new cell through recruitment and differentiation of OPCs (Franklin and Ffrench-Constant, 2008; Wlodarczyk et al., 2017). Here, we focus on the restoration process of the damaged myelin sheath, which is a typical sign of the pathophysiology of MS.

In the case of MS, remyelination is impaired by the presence of inflammation around the nerves and is insufficient to compensate for myelin loss and an unsuccessful differentiation of OPCs. Mechanisms included in this pathology may be associated with a lack of myelination stimulators or molecular inhibitory factors, suppressing OPCs differentiation and myelination (Chari, 2007).

Various molecular factors affect the ability of oligodendrocytes to remyelinate, among which microRNAs (miRNAs) have been shown to play an important regulatory role. These short non-coding RNA molecules act by binding to target mRNAs and inhibiting the process of translation or promoting their degradation. In the biogenesis process, miRNA is transcribed from DNA as a longer precursor, which is then processed by an enzyme complex called Dicer-dependent RNA into a short, double-stranded miRNA molecule. MiRNA molecules interact with proteins in the RNA-induced silence complex (RISC) to form the RISC-miRNA complex, which binds to complementary RNA sequences in the cell. This process results in inhibition of mRNA translation or destabilization, leading to a decrease in the level of protein encoded by mRNA (Gebert and MacRae, 2019). Recent studies have demonstrated that miRNAs are involved in various biological processes, including remyelination in the CNS (Duffy and McCoy, 2020). They operate through the regulation of gene expression related to oligodendrocyte proliferation, myelin production, the process of removing dead or damaged cells, and the expression of genes related to the production of cytokines and chemokines. It appears that inhibition of miRNAs involved in the processing of mature OPCs interrupts normal CNS myelination and that OPCs lacking mature miRNAs are not capable of differentiating properly, as has been shown *in vitro* and *in vivo* (Dugas et al., 2010). The research results also suggest that the specific miRNA restricts neuroinflammation while also promoting remyelination and repair in the CNS after demyelination (Galloway et al., 2019).

In this article, we briefly summarize the biology of remyelination and the current challenges and strategies to induce this process after damage. We focus particular attention on miRNA molecules that are involved in remyelination by regulating gene expression and may be applied in novel diagnostic and therapeutic strategies.

## 2. Remyelination

The degree of remyelination observed between MS lesions is highly variable and depends on the stage of disease progression, the activity of the lesions, and the variation in the underlying pathogenic mechanisms (Patrikios et al., 2006; Wiggermann et al., 2023). Furthermore, the level of remyelination is directly correlated with both the number of oligodendrocytes and macrophages in the lesions (Lucchinetti et al., 1999; Kamma et al., 2022). The process of remyelination involves the migration, proliferation, and differentiation of OPCs that come into contact with the axons, ultimately forming myelin sheaths (Chari, 2007). Moreover, T- and B-cells have also been found to affect the remyelination process. Tregs were shown to promote oligodendrocyte differentiation and remyelination, while Treg-deficient mice have reduced remyelination and differentiation that can be restored by adoptive transfer of Tregs. These cells directly promote the differentiation of OPCs and myelination *in vitro*, and CCN3 was newly identified as a Treg-derived factor that helps with oligodendrocyte differentiation and myelination. These results indicated that Treg cells exhibit a new regenerative role in the CNS that is different from their immunomodulatory function (Dombrowski et al., 2017). Furthermore, the recent study examined how the adoptive transfer of IL-10+ regulatory B cells (Bregs) in female mice with experimental autoimmune encephalomyelitis (EAE) can reverse the disease and promote the expansion of peripheral and CNS-infiltrating IL-10+ T cells. Bregs transfusion resulted in clinical improvement and spinal cord remyelination in EAE Bregs-treated mice, along with the normalization of the immune environment of the CNS and activation of OPCs with subsequent remyelination (Pennati et al., 2020).

In response to demyelination, microglia are activated and migrate to the injury site, where they phagocytose myelin debris and release cytokines and chemokines to attract other cells to the site (Fu et al., 2014). Once the microglia have cleared the debris, the next step is the recruitment and proliferation of OPCs. In the next stage, called the differentiation phase, the OPCs differentiate into pre-myelinating oligodendrocytes, which then contact the demyelinated axons and differentiate into mature, myelinating oligodendrocytes that form functional myelin sheaths (Tepavčević and Lubetzki, 2022). This step is regulated by several molecular signaling pathways, including the Wnt/β-catenin, PI3K/AKT/mTOR, and ERK/MAPK (Gaesser and Fyffe-Maricich, 2016). During remyelination, axons secrete signaling factors such as neuronal growth factor (NGF) and glial cell growth factor (GFAP), which stimulate the migration and differentiation of OPCs. The final step in the remyelination process is the synthesis and assembly of myelin proteins, which are essential for the formation of new myelin sheaths. Myelin proteins are synthesized by mature oligodendrocytes and are assembled into myelin sheaths around axons (Franklin and Ffrench-Constant, 2008). Various proteins and regulatory factors are involved in myelin synthesis, including myelin basic protein (MBP), myelin proteolipid protein (PLP) and myelin-associated glycoprotein (MAG). These proteins bind to myelin, forming insulating layers around axons that prevent the loss of nerve signals (Meschkat et al., 2022).

OPCs are the primary cells responsible for remyelination in the CNS (Bottes and Jessberger, 2021). Molecular markers that identify OPCs or their progeny are useful to detect and quantify remyelination (Valério-Gomes et al., 2018). The transcription factor Olig2 is critical for oligodendrocyte development, but a subset of OPCs in the brain do not express Olig2 throughout life, and this population appears to coincide with changes in brain activity (Fang et al., 2023). Another molecular marker of remyelination is MBP, a component of the myelin sheath. MBP is expressed by mature oligodendrocytes and is down-regulated during demyelination. However, in remyelinating lesions, MBP expression is restored as new myelin sheaths are formed. Detection of MBP expression in tissue sections can be used as an indicator of remyelination (Lindner et al., 2008). In addition to OPCs and myelin components, other molecular markers of remyelination have been identified. One such marker is the transcription factor Sox10, which is expressed in OPCs and their progeny, as well as in Schwann cells (Stolt et al., 2002). Schwann cells are a type of glial cells that normally myelinates peripheral nerves but can also contribute to remyelination in the CNS (Chen et al., 2021). Other molecular markers of remyelination include growth factors and cytokines that regulate the proliferation and differentiation of OPCs. For example, insulin-like growth factor-1 (IGF-1; Mason et al., 2000) and platelet-derived growth factor (PDGF; Woodruff et al., 2004) are important regulators of OPC proliferation and differentiation. These growth factors are up-regulated in remyelinating lesions and can be used as markers of remyelination.

There are several molecular factors that challenge the proper remyelination process during MS (Gruchot et al., 2019). Along with aging and disease duration, the remyelination decreases, which may be due to a decrease in the number of OPCs (Goldschmidt et al., 2009; Hart et al., 2012; Frischer et al., 2015; Neumann et al., 2017). Overactive inflammation, unfavorable microenvironment, the presence of inhibitors and the lack of stimulators can also hinder the process of remyelination (Wang et al., 1998; Charles et al., 2000; Mi et al., 2005; Kuhlmann et al., 2008; Kremer et al., 2019). Researchers are currently investigating various approaches to enhance remyelination in MS (Jolanda Münzel and Williams, 2013; Kremer et al., 2019). These include replacement therapies (Huang and Franklin, 2012), the modifications of stem cells (Uchida et al., 2012), drugs stimulating growth factor production, and immunomodulatory therapies (Kremer et al., 2015) that can reduce inflammation and create a more favorable environment for remyelination. Enhancing endogenous remyelination can be achieved by targeting specific signaling pathways, such as Wnt (Fancy et al., 2009), Neurogenic locus notch homolog (Notch; Aparicio et al., 2013), and Sonic hedgehog (Shh; Loulier et al., 2006), which are involved in the regulation of OPCs differentiation and remyelination. Researchers are also investigating small-molecule therapies that can promote remyelination by stimulating the differentiation of OPCs, improving myelin protein synthesis, or hampering inhibitors of myelination (Medina-Rodríguez et al., 2017). Furthermore, gene therapy is a promising approach that involves the delivery of genetic material to cells to promote remyelination (Billinghurst et al., 1998).

It is likely that a combination of the above approaches will be required to achieve effective remyelination in MS patients.

## 3. MiRNA in remyelination – mechanisms and capabilities

MiRNAs play an essential role in the regulation of the remyelination process at various stages. They act as fine-tuners of gene expression, and their dysregulation has been implicated in various demyelinating diseases such as MS (Dolati et al., 2018).

The use of the EAE mouse model allowed to show that miR-223 is required to efficiently remove myelin debris and promote remyelination (Galloway et al., 2019). miR-223 was needed to efficiently perform the activation and phagocytosis of debris M2 myeloid cells. MiR-233-deficient mice showed impaired CNS remyelination (Galloway et al., 2019). Furthermore, miR-223 has been reported to be dysregulated in myeloid cells from MS patients and to contribute to reparative activation of myeloid cells and remyelination of the CNS (Galloway et al., 2019). In turn, in the murine BV_2_ microglia cells, miR-155-3p has been shown to be up-regulated in response to demyelination and to promote microglia activation and increase the production of pro-inflammatory mediators, such as tumor necrosis factor (TNF)-α, interleukins (IL-1, IL-6) and nitric oxide (NO), through the negative regulation of the SOCS1 signaling pathway (Zheng et al., 2018). Although microglial activation is a key step in initiating the remyelination process, its chronic and uncontrolled activation can lead to neurotoxic effects (Liu et al., 2013). Therefore, miR-155-3p has appeared to be a good target for restoring dysfunctional microglia and promoting myelination (Butovsky et al., 2015).

Transformation of OPCs to myelinating oligodendrocytes is a complex process, greatly influenced by transcription factors, particularly the Sox protein family (Stolt et al., 2002, 2003, 2006). Recent research has identified miR-204 as a new target gene of Sox10, a critical regulator in the development of oligodendroglia (Stolt et al., 2006). MiR-204 has been found to suppress OPC proliferation and promote oligodendrocyte differentiation by inhibiting pro-proliferative Ccnd2 and differentiation-inhibiting Sox4 (Wittstatt et al., 2020). The study by Wittstatt et al. (2020) shows that Sox10 plays a key role in driving oligodendroglial cells to exit the cell cycle and initiate differentiation through miR-204 induction (Wittstatt et al., 2020).

The improper balance of the components of the RISC complex in oligodendrocytes has been shown to result in decreased levels of miRNAs that are crucial for oligodendrocyte differentiation, survival, and myelin synthesis: miR-106b-5p, −15a-5p, −15b-5p, −181a-5p, −181c-5p, −181d-5p, −20b-5p, −320-3p, −328-3p, −338-3p, −20a, and −92a-1. Meanwhile, the formation of abnormal RISC in T-cells that infiltrate the brain contributed to the polarization of miRNA-dependent proinflammatory helper T (Th)-cells. Based on the research findings, it is suggested that the dysregulation of miRNA in EAE/MS might be caused by the defective assembly of RISC, which allows autoreactive effector T-cells to maintain a highly specific proinflammatory program (Lewkowicz et al., 2015). MiR-92a has also been reported to drive autoimmunity in the CNS by maintaining the imbalance of the regulatory T (Treg)/Th17 ratio in MS patients (Fujiwara et al., 2022). Moreover, a new subpopulation of myelin-specific CD49d + CD154+ lymphocytes present in the peripheral blood of MS patients during remission were found to have the unique ability to migrate to maturing OPCs and synthesize proinflammatory chemokines/cytokines, which interferes with the remyelination process (Piatek et al., 2020). The presence of myelin-specific CD49d + CD154+ lymphocytes close to maturing OPCs and remyelinating plaques has been confirmed in mice during disease remission. CD49d + CD154+ cells affected the maturation of OPCs toward immune reactive oligodendrocytes, which were characterized by uneven production of MBP and PLP and pro-inflammatory mediators. The examination of cellular pathways responsible for reprogramming of the oligodendrocytes revealed that CD49d + CD154+ lymphocytes had an impact on miRNA production by disrupting polymerase II activity. CD49d + CD154+ targeted miR-665 and ELL3 and when the high level of miR-665 was neutralized, miRNA and MBP/PLP synthesis were restored (Piatek et al., 2019).

Furthermore, miR-219 has been shown to rapidly promote OPCs differentiation in mature oligodendrocytes by targeting the expression of platelet-derived growth factor receptor (PDGFR)α, Sox6, FoxJ3, and zinc finger protein (ZFP) 238, which result in inhibition of OPCs proliferation and pass to the oligodendrocyte differentiation phase (Dugas et al., 2010). Inhibition of miR-219 strongly affects the differentiation of oligodendrocytes, while miR-219 alone can instigate oligodendrocytes differentiation in OPCs immersed in mitogens and can also partially rescue the differentiation deficit caused by the loss of mature miRNA production in oligodendrocytes (Dugas et al., 2010). Overexpression of miR-219 has been found to promote precocious oligodendrocyte maturation and regeneration processes in mice. The study also identified a network for miR-219 targeting of differentiation inhibitors, including Lingo1 and Etv5, and inhibition of these factors partially rescued differentiation defects of miR-219-deficient oligodendrocyte precursors (Wang H. et al., 2017).

By selectively deleting the miRNA processing enzyme Dicer1 in oligodendrocyte lineage cells, it was found that mice lacking Dicer1 had severe myelinating deficits despite the expansion of the oligodendrocyte progenitor pool. Further experiments identified miR-219 and miR-338 as oligodendrocyte-specific miRNAs that promote oligodendrocyte differentiation by repressing negative regulators of oligodendrocyte differentiation, including transcription factors Sox6 and Hes5 (Zhao et al., 2010). Based on these findings, a study including cerebrospinal fluid (CSF) miR-219 obtained from MS patients has been carried out. The lack of detection of CSF miR-219 has been associated with MS in all three cohorts of patients (RRMS, SPMS, PPMS) compared to controls (Bruinsma et al., 2017).

The effect of miR-125a-3p on oligodendroglial maturation in cultured OPCs has also been studied. It has been suggested that over-expression of miR-125a-3p impairs maturation, while inhibiting it stimulates maturation. The abnormally high levels of miR-125a-3p in the CSF of MS patients with active demyelinating lesions have been reported. This miRNA molecule was also upregulated in active lesions of MS patients and in OPCs isolated from the spinal cord of EAE. The study identified Slc8a3 and Gas7 as targets of miR-125a-3p, with Gas7 necessary for correct oligodendrocyte terminal maturation (Marangon et al., 2020). This suggests that the overexpression of miR-125a-3p may contribute to the development of MS by blocking OPC differentiation, which impairs the repair of demyelinated lesions (Lecca et al., 2016).

Another study conducted in rats showed that miR-212 level was reduced at the spinal cord injury site after injury and that it is expressed in oligodendrocytes and glial progenitor cells in the adult CNS. The researchers found that reducing the expression of miR-212 improved the cell process outgrowth of oligodendrocytes and up-regulated genes associated with their differentiation and maturation, including OLIG1, SOX10, MBP, and PLP1. On the other hand, increased expression of miR-212 in glial progenitor cells or OPCs decreased the expression of these genes. The study also showed that PLP1 is a direct target molecule of miR-212 and that its overexpression inhibited oligodendrocyte maturation-associated proteins, including 2′, 3′-cyclic nucleotide 3′-phosphodiesterase (CNPase), MBP, and PLP, and the oligodendrocyte extension process (Wang C.-Y. et al., 2017).

A study by Morris et al. (2015) suggested the role of neuronal miRNA, miR-124, in controlling gene expression in oligodendrocytes. Loss of miR-124 resulted in a decrease in the number of oligodendrocyte cells and myelination of axonal projections in the ventral hindbrain model of zebrafish embryos (*Danio rerio*) model. When miR-124 levels were reduced, there was a decrease in the number of MBP-positive oligodendrocytes and MBP RNA did not pass through the oligodendrocyte processes (Morris et al., 2015).

The study by Tripathi et al. (2019) demonstrated that increased levels of miR-27a were found in OPCs associated with MS lesions and in animal models of demyelination. Increased levels of miR-27a led to inhibition of OPCs proliferation and impaired differentiation of human OPCs by dysregulating the Wnt-β-catenin signaling pathway. Administration of miR-27a led to suppression of myelinogenic signals, resulting in loss of endogenous myelination and remyelination. These findings suggest that a steady-state level of oligodendrocyte-specific miR-27a is critical in supporting multiple steps in the complex process of OPCs maturation and remyelination (Tripathi et al., 2019).

Interestingly, miR-297c-5p has been found to play a dual role in remyelination. It acts as a negative regulator of OPCs proliferation and a positive regulator of oligodendrocyte maturation by targeting cyclin T2 (CCNT2), the regulatory subunit of positive transcription elongation factor b, which inhibits oligodendrocyte maturation. When miR-297c-5p was overexpressed in mouse embryonic fibroblasts and rat OPCs, it promoted G1/G0 arrest and increased the number of O1+ rat OPCs during differentiation. These findings suggest that miR-297c-5p may be a potential target for promoting oligodendrocytes maturation and enhancing remyelination in diseases with a demyelinating component (Kuypers et al., 2016).

The role of miR-23a in enhancing oligodendrogenesis and myelin synthesis *in vivo* has been analyzed. Previous research showed that miR-23 can enhance oligodendrocytes differentiation and that lamin B1 negatively regulates oligodendrocytes (Lin and Fu, 2009). The study used mice in which miR-23a is overexpressed by an oligodendrocyte-specific promoter to investigate the effects of miR-23a. It has been found that miR-23a modulates the expression of phosphatase and tensin homolog on chromosome 10 (PTEN) and a long noncoding RNA (lncRNA) – 2700046G09Rik, which fine-tunes activities of the Akt/mTOR and MAPK pathways, promoting myelin gene expression. The results suggest that myelination requires tightly regulated multilayer signaling pathways (Lin et al., 2013).

In the attachment and extension of oligodendrocyte processes, miR-219 has been shown to be involved by promoting the expression of integrins and adhesion molecules. It has been reported that the myelination defects observed *in vitro* and *in vivo* are directly caused by the loss of Dicer1 function in OPCs and mature oligodendrocytes, resulting in ineffective action of miRNA (Dugas et al., 2010). Furthermore, studies on Dicer-floxed mice specific for oligodendrocytes showed that miR-219 targeting the elongation of the very long chain fatty acid protein (ELOVL7) plays a role in the maintenance of lipids and redox homeostasis in mature oligodendrocytes, required for the formation and integrity of myelin (Shin et al., 2009).

In the synthesis and assembly of myelin proteins, several miRNAs have been shown to be involved, including miR-138, miR-145, and miR-338. These miRNAs promote the expression of myelin genes by targeting negative regulators of the cAMP signaling pathway and the epigenetic regulator HDAC2. The overexpression of miR-146a in primary OPCs increased their expression of myelin proteins, while the reduction of endogenous miR-146a levels inhibited the generation of these proteins (Liu et al., 2017). The study also found that miR-146a inversely regulated the expression of its target gene-IRAK1 in OPCs, and suppressing the expression of IRAK1 in OPCs significantly increased myelin proteins and decreased OPC apoptosis (Liu et al., 2017).

**Figure 1:**
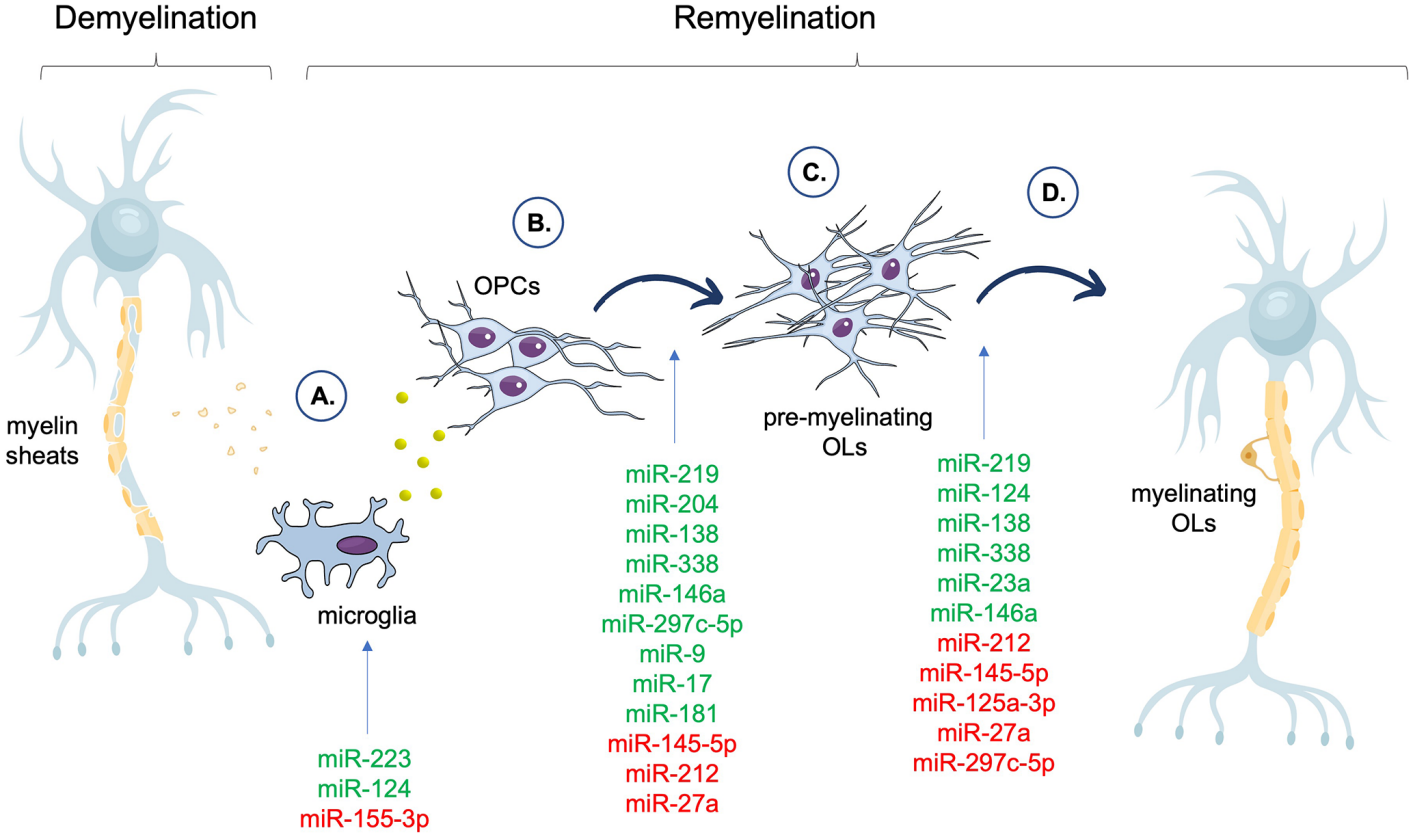
Summary of the miRNAs associated with different phases of the remyelination process. In the figure, miRNAs that have a positive impact on a particular stage of remyelination are shown in green, whereas miRNAs that have a negative effect on a particular stage of remyelination are shown in red. **(A)** Clearance of myelin debris and secreted of factors by microglia. **(B)** OPCs recruitment and proliferation. **(C)** OPCs differentiation and maturation. **(D)** Myelin proteins assembly. OPCs, Oligodendrocyte Precursor Cells (OPCs); OLs, oligodendrocytes.

The contribution of selected miRNAs to the particular stages of remyelination is summarized in the Figure 1.

## 4. The concept of miRNA as a biomarker for MS

In addition to the usefulness of miRNAs as candidates for the development of novel therapeutic strategies for remyelination, they have also been proposed as biomarkers for the diagnosis and prognosis of MS (Regev et al., 2016). While miRNA profiling from biological fluid poses great promise as a biomarker for MS, it must be noted that often conflicting results, high heterogeneity, and lack of repeatability are becoming challenging in the field (Piket et al., 2019). However, the lack of compliance is not unfamiliar to all biomarker projects and underscores only the logistical difficulties in such kind of research (Zurawska et al., 2019).

Currently used biomarkers for MS diagnosing are insufficient in terms of sensitivity and specificity, and prevent recognizing patients who are in the asymptomatic phase of MS, prior to the onset of clinical manifestations. In MS, the disease progression and the presence of active inflammation within the brain are usually monitored by gadolinium (Gd) enhanced magnetic resonance imaging (MRI) technique. Muñoz-San Martín et al. (2019) showed a positive correlation between miR-21, miR-146a, and miR-146b upregulation in CSF from MS patients with Gd + lesions suggesting that analyzed miRNAs are useful biomarkers in identifying the active lesions (Muñoz-San Martín et al., 2019). Selmaj et al. (2017) isolated exosomes from serum from RRMS patients (33 with relapse and 30 with remission) and a control group. Specimens from all patients were sampled before methylprednisolone administration, and patients in remission did not receive DMT for at least 6 months. Using the qPCR technique demonstrated that miR-122-5p, miR-196b-5p, and miR-532-5p were significantly downregulated in relapse patients compared to remission patients. Furthermore, by ROC analysis, a combination of miR-122-5p and miR-196b-5p gave AUC of 0.866 for distinguishing RRMS relapse from RRMS remission. While lower levels of miR-122-5p, miR-196b-5p, miR-301a-3p, and miR-532-5p were related to disease activity (Selmaj et al., 2017). Regev et al. (2018) reported 5 miRNAs (miR-484, miR-140-5p, miR-320a, miR-486-5p, and miR-320c), which demonstrated an essential difference between MS patients and the control group, according to data from 4 cohort studies (Regev et al., 2018). Other study performed on CSF from 53 MS patients and 39 healthy volunteers demonstrated that miR-181c, miR-633, and miR-922 were specifically expressed only in MS patients (Haghikia et al., 2012). A large-scale study by Vistbakka et al. (2018) showed that miR-191-5p and miR-24-3p were significantly up-regulated in both RRMS and PPMS compared to the control group (Vistbakka et al., 2018). Another large-scale study by Nuzziello et al. (2018) that included whole blood samples from 58 MS patients (54 RRMS and 4 SPMS), and 20 healthy controls reported that miR-320a, miR-125a-5p, miR-652-3p, miR-185-5p, miR-942-5p, and miR-25-3p were significantly upregulated in MS compared to the control group. The area under the curve (AUC) values for validated miRNAs ranged from 0.701 to 0.735 and are fair tests to discriminate MS patients from controls, with miR-320a having the highest AUC value (0.735) (Nuzziello et al., 2018).

In addition, there are few studies that miRNA profiling is a useful tool to identify MS subtypes. A cohort study on a large group of MS patients (*n* = 1,088) was based on an examination of the relationship between MRI brain imaging and miRNA profiling. Surprisingly, each MRI-phenotypes (different in terms of brain atrophy and cerebral T2-hyperintense lesion volume) was linked with a characteristic miRNA signature, especially miR-22-3p, miR-361-5p, and miR-345-5p, which were the most valid differentiators of the MRI-phenotypes in MS patients (Hemond et al., 2019). Ebrahimkhani et al. (2017) using NGS identified 9 miRNAs dysregulated in RRMS compared to progressive MS patients (SPMS/PPMS) (6 upregulated: miR-15b-5p, miR-23a-3p, miR-223-3p, miR-30b-5p, miR-342-3p, miR-374a-5p, and 3 downregulated: miR-432-5p, miR-433-3p, miR-485-5p). Furthermore, ROC analysis allowed distinguishing RRMS from SPMS/PPMS patients with AUC values for miR-433-3p, miR-432-5p, and miR-485-5p were 0.93, 0.86, and 0.87, respectively (Ebrahimkhani et al., 2017). The recent study by Edgünlü et al. (2022) showed that the expression of miR-181a-5p was downregulated and associated with an increased risk of MS (*p* = 0.012). Furthermore, *in silico* analyses showed that circulating miR-181a-5p can participate in MS pathology by targeting genes involved in inflammation and neurodegeneration molecular pathways, such as MAP2K1, CREB1, ATXN1, and ATXN3 (Edgünlü et al., 2022).

## 5. MiRNA in novel therapeutic and diagnostic strategies of remyelination – facts and expectations

Although there are various treatments available that aim to decrease the immune response in MS, currently there is no treatment that encourages the regeneration of myelin. The disease-modifying therapies (DMTs) are a key component of MS symptoms management; however, the effects of these treatments vary depending on the individual and the type of MS. Moreover, they may not provide complete symptom management and can lead to significant side effects. They are also insufficient to prevent the accumulation of permanent disability caused by neurodegeneration, especially in the progressive phase of the disease (Goldenberg, 2012). As a result, alternative approaches are emerging. The identification of miRNAs as regulators of the remyelination process has led to the development of novel therapeutic strategies targeting these molecules.

Direct administration of miRNAs to the CNS is currently difficult and invasive, thus, to overcome this issue, various delivery systems, including poly (lactic-co-glycolic acid) (PLGA) nanoparticles, liposomes, and extracellular vesicles (EVs) have been developed. These delivery systems can protect miRNA from degradation and facilitate its uptake by oligodendrocytes and other cells in the CNS. The research results suggest that EVs hold the most promise as a non-invasive and efficient delivery system for miRNAs, as they were able to induce remyelination in an animal model. Moreover, it has been reported that miR-219a-5p encapsulated in EVs stimulate OPCs differentiation, can cross the blood–brain barrier (BBB) and improve the clinical transformation of EAE (Osorio-Querejeta et al., 2020). These findings seem promising in the context of a novel therapeutic approach for MS patients.

Interestingly, a recent study investigated the effect of exposure of aging rats to a young systemic environment on the production of serum exosomes involved in the promotion of remyelination by increasing the number and differentiation of OPCs. It has been shown that the so-called environmental enrichment (social, cognitive, and physical exposition to the youth environment) of aging animals stimulates the production of exosomes that mimic the promyelinating effect (Pusic and Kraig, 2014). Environmental enrichment has previously been shown to improve memory and myelin production, alleviate the consequences of neurodegeneration (Fields, 2008), enhance immune system functioning (Pedersen and Hoffman-Goetz, 2000), and reduce oxidative stress (Arranz et al., 2010). Exosomes derived from both young and environmental enrichment animals were enriched in miR-219 molecules. The nasal administration of exosomes to aging rats improved myelination, which may be useful in novel remyelination strategies (Pusic and Kraig, 2014). Further studies support findings regarding the role of miR-219 in the pro-myelinating effect. Moreover, in this study, exosomal miR-9, miR-17, and miR-181 have been revealed to contribute to oligodendrocyte proliferation and anti-inflammatory process (Pusic et al., 2016).

The study by Li et al. aimed to investigate the therapeutic effects of M2 microglia-derived EVs in promoting white matter repair and functional recovery after cerebral ischemia in mice. The researchers found that M2-EVs treatment led to increased oligodendrogenesis and white matter repair, resulting in improved functional recovery. The therapeutic effect was attributed to the presence of miRNAs, including miR-23a-5p, miR-221-3p, miR-129-5p, and miR-155-5p, in M2-EVs, which promoted the survival and differentiation of OPCs. In particular, miR-23a-5p was identified as a key miRNA that promoted OPC differentiation by targeting Olig3 directly (Li et al., 2022).

Another study on potential miRNA delivery tools investigated the utility of using a biodegradable and biocompatible cationic polymer called PDAPEI to deliver miRNAs for therapeutic purposes. The study found that PDAPEI was less toxic and more efficient in delivering miR-221/222 to rat Schwann cells than another polymer called PEI25kDa. Upregulation of miR-221/222 in Schwann cells promoted the expression of NGF and MBP. The study also tested the effectiveness of PDAPEI/miR-221/222 complexes in promoting nerve regeneration in a mouse sciatic nerve crush injury model. The results showed that the complexes significantly enhanced remyelination and promoted nerve regeneration. In general, the study suggests that PDAPEI/miR-221/222 complexes may provide a safe and effective means of treating nerve crush injury (Song et al., 2017).

The next proposed platform to promote remyelination in the CNS was a scaffolding system for sustained nonviral delivery of miRNAs to promote the differentiation, maturation, and myelination of oligodendrocytes. The miRNAs were incorporated into a fiber-hydrogel scaffold. It has been found to promote the differentiation and myelination of oligodendrocytes *in vitro* and *in vivo* after spinal cord injury in rats. The miR-219/miR-338 treatment increased the number of oligodendrocytes and the rate and extent of their differentiation, resulting in more compact myelin sheaths and higher myelination (Milbreta et al., 2019).

Another study aimed to understand the molecular pathway by which NGF negatively regulates oligodendrogenesis by investigating downstream targets, focusing on miRNAs. The study used a mouse model deprivation of NGF and found that NGF inhibits oligodendrogenesis by negatively regulating the expression of miR-219a-5p, which is a positive regulator of oligodendrocyte differentiation and myelin repair. These findings suggest that NGF can be targeted to enhance myelination and promote remyelination in demyelinating diseases such as MS (Brandi et al., 2021).

As mentioned before, miR-223 expression is essential for the efficient clearing of myelin debris after demyelination and is upregulated in active MS lesions, likely due to macrophage infiltration and proliferation (Galloway et al., 2019). The NLR family pyrin domain containing 3 (NLRP3) inflammasome and miR-223-3p are up-regulated immediately after demyelination and returned to near baseline after remyelination (Galloway et al., 2022). The NLRP3 inflammasome is a protein complex that is part of the innate immune system, which is responsible for initiating the body’s inflammatory response to potential threats, such as pathogens and tissue damage (Swanson et al., 2019). It has been revealed that the NLRP3 inflammasome was primarily expressed within activated macrophages/microglia, both in experimentally induced demyelination and mixed active/inactive MS lesions. *In vitro*, the small-molecule NLRP3 inhibitor, MCC950, and miR-223-3p mimics suppressed the activation of the NLRP3 inflammasome in macrophages and microglia. When delivering MCC950 to animals after lysolecithin-induced demyelination, the axonal injury within the demyelinated lesions was significantly reduced. The results suggest that the NLRP3 inflammasome plays a role in demyelinating injury and that NLRP3 inhibitors may serve as an effective new therapeutic strategy for treating MS (Galloway et al., 2022).

Otero-Ortega et al. (2017) made initial observations from deep RNA sequencing data of exosomes derived from mesenchymal stem cells (MSCs) and hypothesized that certain miRNAs, such as miR-199a-5p and miR-145, could be involved in oligodendrocyte maturation. In a model of subcortical ischemic stroke, MSC-derived exosomes were found to facilitate the differentiation of oligodendrocytes and the remyelination process. Upon intravenous administration, the authors observed increased levels of myelin protein and a greater number of myelinated axons (Otero-Ortega et al., 2017). These findings were consistent with the results of an *in vitro* model of ischemic stroke, which demonstrated that miR-134, obtained from bone marrow MSC exosomes, exerted a positive impact on rat oligodendrocytes by suppressing apoptosis by targeting caspase-8 (Xiao et al., 2019).

One study investigated the impact of hippocampal demyelination on neuronal gene expression and memory impairment in MS patients. Comparative analysis of miRNA profiles from myelinated and demyelinated hippocampi from the postmortem brain of MS revealed that demyelination led to increased expression of miR-124, which targets mRNAs encoding several neuronal proteins, including α-amino-3-hydroxy-5-methyl-4-isoxazolepropionic acid (AMPA2) and AMPA3 receptors. It has been also observed that hippocampal demyelination in mice increased miR-124, reduced expression of AMPA receptors, and decreased memory performance in water maze tests. However, remyelination of the mouse hippocampus reversed these changes (Dutta et al., 2013).

A recent study investigated the effect of Hydroxychloroquine, an antimalarial immunomodulatory medication, on microglia and oligodendrocytes by regulating the expression of miR-219 and miR-155-3p in the cuprizone-induced demyelination mice model (Mazloumfard et al., 2020). The influence of Hydroxychloroquine on the activity of microglia and/or oligodendrocytes has previously been established (Koch et al., 2015). The study revealed that pharmacological strategies leading to miR-155-3p down-regulation may enhance remyelination in MS (Mazloumfard et al., 2020).

Another study on miR-219 and miR-155-3p expression levels has been carried out in the context of myelination with the use of Apamin in a cuprizone-induced demyelination mice model. Apamin exhibited more impact on the reduction in miR-155-3p expression in the demyelination phase of the disease than the elevation of miR-219 in the remyelination phase and has been suggested as a therapeutic option to reduce plaque formation during the exacerbation phase of MS by reducing the expression of miR-155-3p (Gholami et al., 2020).

The common effect of the action of miR-219 and miR-338 to promote OPCs differentiation, maturation, and myelination may serve as a promising strategy for nerve repair, as has been reported using the scratch test, which recreated a nerve injury *in vitro* (Nguyen et al., 2019). A study by Diao et al. aimed to improve oligodendrocyte differentiation and maturation by developing a nanofiber-mediated miRNA delivery method to control the differentiation of OPCs through a combination of fiber topography and gene silencing. The study showed that nanofiber topography enhanced OPCs differentiation, while miRNA delivery further improved the results. Furthermore, nanofiber-mediated delivery of miR-219 and miR-338 promoted the maturation of oligodendrocytes. The study’s results demonstrate the potential of nanofibers in providing topographical cues and miRNA delivery to direct OPCs differentiation and may find useful applications in treating CNS pathological conditions that require remyelination (Diao et al., 2015).

The use of miRNAs as biomarkers of remyelination in MS provided several advantages. Firstly, these molecules can be detected in easily accessible biofluids, such as CSF and blood, making them attractive as non-invasive biomarkers (McCoy, 2017). Furthermore, miRNAs are stable and can be reliably detected using common quantitative real-time PCR (qPCR), microarrays or next-generation sequencing (NGS) techniques (Moody et al., 2017). Then, differences in the expression of certain miRNAs seem to be specific to some biological and pathological processes and therefore can provide more accurate information about the disease process than other biomarkers (Sheinerman et al., 2017). Despite this, several challenges hamper their application into clinical diagnostic practice, such as the variability in miRNA expression across individuals, the lack of standardization in miRNA detection methods, and data normalization, which affect the accuracy and reproducibility of results (Piket et al., 2019).

One study aimed to identify miRNA expression patterns during the maturation of oligodendrocytes from human embryonic stem (hES) cells. The miRNA analysis in cells from eight stages of oligodendrocytes differentiation has been performed. MiRNA expression patterns have been found to be similar to those in rat and mouse CNS cells, with four distinct clusters of miRNA expression corresponding to different stages of oligodendrocyte maturation. The study also identified potential mRNA targets for these miRNAs, including factors involved in oligodendrocyte development and myelination, such as C11Orf9, CLDN11, MYTL1, MBOP, MPZL2, and DDR1. These findings provide insights into the molecular mechanisms of oligodendrocyte differentiation and may serve as markers for oligodendrocytes maturation (Letzen et al., 2010).

## 6. Conclusion

Over the last few years, significant advances have been made in understanding how miRNAs control gene expression post-transcriptionally to regulate CNS myelination. Studies indicate that miRNAs do not act alone, but rather influence multiple signaling and regulatory pathways, which may affect their effectiveness as therapeutic targets. Therefore, it is necessary to understand more complex regulatory mechanisms before miRNAs can be used to treat demyelinating diseases. The miRNA molecule miR-219 is highly expressed in mature myelinating oligodendrocytes and plays a crucial role in promoting the differentiation of precursor oligodendrocyte cells into mature oligodendrocytes. Reduced levels of miR-219 have been observed in MS patients, which can contribute to failed remyelination. Increasing miR-219 has been shown to enhance the maturation of oligodendrocyte precursor cells and is a promising target for remyelination. The use of drugs to stimulate myelin restoration in the CNS could greatly benefit patients by slowing or even protecting against neurodegeneration. However, most of the studies are developed in animal models, which retains several barriers before introducing remyelination strategies into clinical practice. Various models have been created to study inflammatory demyelinating diseases in animals, including immunization, virus-induced, genetic, and toxic models (Ransohoff, 2012). Nevertheless, none of them perfectly replicates the specific characteristics of MS lesions, complexity of the disease pathophysiology, integrating immune and nervous system, contribution of distinct environmental factors, role of T CD8+ cells, mechanisms of the disease progression and age-dependency (Lassmann and Bradl, 2017). Observing remyelination in human samples is difficult due to the limited access to histopathological material and insufficient reflection of remyelination processes in biomarkers derived from body fluids. EAE is, so far, the most commonly employed animal model, as it is able to reflect immune response, inflammation, demyelination, axonal loss, gliosis, and remyelination. Despite critical comments, studies often show that the results obtained from EAE and MS are comparable and are an integral tool in conducting MS research on aspects of autoimmunity, neuroinflammation, and neuronal loss (Birmpili et al., 2022). It should be borne in mind, first of all, that any hypothesis verified on an animal model must be tested on patient material and in clinical trials, which will be its only final confirmation.

Remyelination is believed to prevent progressive axonal injury and reduce long-term disability in MS patients. Therefore, there is a clear need for therapeutic approaches that can enhance the organism’s own repair and remyelination mechanisms.

## Author contributions

All authors contributed to the conception of the work, drafted and revised the manuscript.

## Conflict of interest

The authors declare that the research was conducted in the absence of any commercial or financial relationships that could be construed as a potential conflict of interest.

## Publisher’s note

All claims expressed in this article are solely those of the authors and do not necessarily represent those of their affiliated organizations, or those of the publisher, the editors and the reviewers. Any product that may be evaluated in this article, or claim that may be made by its manufacturer, is not guaranteed or endorsed by the publisher.

## References

[ref1] Aparicio E. Mathieu P. Pereira Luppi M. Almeira Gubiani M. F. Adamo A. M. (2013). The notch signaling pathway: its role in focal CNS demyelination and apotransferrin-induced remyelination. J. Neurochem. 127, 819–836. doi: 10.1111/jnc.12440, PMID: 24032544

[ref2] Arranz L. De Castro N. M. Baeza I. Maté I. Viveros M. P. De la Fuente M. (2010). Environmental enrichment improves age-related immune system impairment: long-term exposure since adulthood increases life span in mice. Rejuvenation Res. 13, 415–428. doi: 10.1089/rej.2009.0989, PMID: 20707722

[ref3] Atlas of MS 2020 – Epidemiology Report (2020). MS International Federation. Available at: https://www.msif.org/resource/atlas-of-ms-2020/ (accessed March 28, 2023).

[ref4] Billinghurst L. L. Taylor R. M. Snyder E. Y. (1998). Remyelination: cellular and gene therapy. Semin. Pediatr. Neurol. 5, 211–228. doi: 10.1016/s1071-9091(98)80036-3 9777679

[ref5] Birmpili D. Charmarke Askar I. Bigaut K. Bagnard D. (2022). The translatability of multiple sclerosis animal models for biomarkers discovery and their clinical use. Int. J. Mol. Sci. 23:11532. doi: 10.3390/ijms231911532, PMID: 36232832 PMC9570245

[ref6] Bottes S. Jessberger S. (2021). Live imaging of remyelination in the adult mouse corpus callosum. Proc. Natl. Acad. Sci. 118:e2025795118. doi: 10.1073/pnas.2025795118, PMID: 34244440 PMC8285919

[ref7] Brandi R. Fabiano M. Giorgi C. Arisi I. La Regina F. Malerba F.. (2021). Nerve growth factor neutralization promotes oligodendrogenesis by increasing miR-219a-5p levels. Cells 10:405. doi: 10.3390/cells10020405, PMID: 33669304 PMC7920049

[ref8] Bruinsma I. B. van Dijk M. Bridel C. van de Lisdonk T. Haverkort S. Q. Runia T. F.. (2017). Regulator of oligodendrocyte maturation, miR-219, a potential biomarker for MS. J. Neuroinflammation 14:235. doi: 10.1186/s12974-017-1006-3, PMID: 29202778 PMC5716023

[ref9] Butovsky O. Jedrychowski M. P. Cialic R. Krasemann S. Murugaiyan G. Fanek Z.. (2015). Targeting miR-155 restores abnormal microglia and attenuates disease in SOD1 mice. Ann. Neurol. 77, 75–99. doi: 10.1002/ana.24304, PMID: 25381879 PMC4432483

[ref10] Chari D. M. (2007). Remyelination in multiple sclerosis. Int. Rev. Neurobiol. 79, 589–620. doi: 10.1016/S0074-7742(07)79026-8, PMID: 17531860 PMC7112255

[ref11] Charles P. Hernandez M. P. Stankoff B. Aigrot M. S. Colin C. Rougon G.. (2000). Negative regulation of central nervous system myelination by polysialylated-neural cell adhesion molecule. Proc. Natl. Acad. Sci. U. S. A. 97, 7585–7590. doi: 10.1073/pnas.100076197, PMID: 10840047 PMC16589

[ref12] Chen C. Z. Neumann B. Förster S. Franklin R. J. M. (2021). Schwann cell remyelination of the central nervous system: why does it happen and what are the benefits? Open Biol. 11:200352. doi: 10.1098/rsob.200352, PMID: 33497588 PMC7881176

[ref13] Dendrou C. A. Fugger L. Friese M. A. (2015). Immunopathology of multiple sclerosis. Nat. Rev. Immunol. 15, 545–558. doi: 10.1038/nri3871 26250739

[ref14] Diao H. J. Low W. C. Milbreta U. Lu Q. R. Chew S. Y. (2015). Nanofiber-mediated microRNA delivery to enhance differentiation and maturation of oligodendroglial precursor cells. J. Control. Release 208, 85–92. doi: 10.1016/j.jconrel.2015.03.005, PMID: 25747407 PMC4779954

[ref15] Dolati S. Marofi F. Babaloo Z. Aghebati-Maleki L. Roshangar L. Ahmadi M.. (2018). Dysregulated network of miRNAs involved in the pathogenesis of multiple sclerosis. Biomed. Pharmacother. 104, 280–290. doi: 10.1016/j.biopha.2018.05.050, PMID: 29775896

[ref16] Dombrowski Y. O’Hagan T. Dittmer M. Penalva R. Mayoral S. R. Bankhead P.. (2017). Regulatory T cells promote myelin regeneration in the central nervous system. Nat. Neurosci. 20, 674–680. doi: 10.1038/nn.4528, PMID: 28288125 PMC5409501

[ref17] Duffy C. P. McCoy C. E. (2020). The role of MicroRNAs in repair processes in multiple sclerosis. Cells 9:1711. doi: 10.3390/cells9071711, PMID: 32708794 PMC7408558

[ref18] Dugas J. C. Cuellar T. L. Scholze A. Ason B. Ibrahim A. Emery B.. (2010). Dicer1 and miR-219 are required for normal oligodendrocyte differentiation and myelination. Neuron 65, 597–611. doi: 10.1016/j.neuron.2010.01.027, PMID: 20223197 PMC2843397

[ref19] Dutta R. Chomyk A. M. Chang A. Ribaudo M. V. Deckard S. A. Doud M. K.. (2013). Hippocampal demyelination and memory dysfunction are associated with increased levels of the neuronal microRNA miR-124 and reduced AMPA receptors. Ann. Neurol. 73, 637–645. doi: 10.1002/ana.23860, PMID: 23595422 PMC3679350

[ref20] Ebrahimkhani S. Vafaee F. Young P. E. Hur S. S. J. Hawke S. Devenney E.. (2017). Exosomal microRNA signatures in multiple sclerosis reflect disease status. Sci. Rep. 7:14293. doi: 10.1038/s41598-017-14301-3, PMID: 29084979 PMC5662562

[ref21] Edgünlü T. G. Yılmaz Ş. G. Emre U. Taşdelen B. Kuru O. Kutlu G.. (2022). miR-181a-5p is a potential candidate epigenetic biomarker in multiple sclerosis. Genome 65, 547–561. doi: 10.1139/gen-2022-0040, PMID: 36103723

[ref22] Engelhardt B. Ransohoff R. M. (2005). The ins and outs of T-lymphocyte trafficking to the CNS: anatomical sites and molecular mechanisms. Trends Immunol. 26, 485–495. doi: 10.1016/j.it.2005.07.004, PMID: 16039904

[ref23] Fancy S. P. J. Baranzini S. E. Zhao C. Yuk D.-I. Irvine K.-A. Kaing S.. (2009). Dysregulation of the Wnt pathway inhibits timely myelination and remyelination in the mammalian CNS. Genes Dev. 23, 1571–1585. doi: 10.1101/gad.1806309, PMID: 19515974 PMC2704469

[ref24] Fang L.-P. Liu Q. Meyer E. Welle A. Huang W. Scheller A.. (2023). A subset of OPCs do not express Olig2 during development which can be increased in the adult by brain injuries and complex motor learning. Glia 71, 415–430. doi: 10.1002/glia.24284 36308278

[ref25] Fields R. D. (2008). White matter in learning, cognition and psychiatric disorders. Trends Neurosci. 31, 361–370. doi: 10.1016/j.tins.2008.04.001, PMID: 18538868 PMC2486416

[ref26] Filippi M. Bar-Or A. Piehl F. Preziosa P. Solari A. Vukusic S.. (2018). Multiple sclerosis. Nat. Rev. Dis. Primers. 4, 1–27. doi: 10.1038/s41572-018-0041-4 30410033

[ref27] Franklin R. J. M. Ffrench-Constant C. (2008). Remyelination in the CNS: from biology to therapy. Nat. Rev. Neurosci. 9, 839–855. doi: 10.1038/nrn2480 18931697

[ref28] Frischer J. M. Weigand S. D. Guo Y. Kale N. Parisi J. E. Pirko I.. (2015). Clinical and pathological insights into the dynamic nature of the white matter multiple sclerosis plaque. Ann. Neurol. 78, 710–721. doi: 10.1002/ana.24497, PMID: 26239536 PMC4623970

[ref29] Fu R. Shen Q. Xu P. Luo J. J. Tang Y. (2014). Phagocytosis of microglia in the central nervous system diseases. Mol. Neurobiol. 49, 1422–1434. doi: 10.1007/s12035-013-8620-6, PMID: 24395130 PMC4012154

[ref30] Fujiwara M. Raheja R. Garo L. P. Ajay A. K. Kadowaki-Saga R. Karandikar S. H.. (2022). microRNA-92a promotes CNS autoimmunity by modulating the regulatory and inflammatory T cell balance. J. Clin. Invest. 132:e155693. doi: 10.1172/JCI155693, PMID: 35298438 PMC9106347

[ref31] Gaesser J. M. Fyffe-Maricich S. L. (2016). Intracellular signaling pathway regulation of myelination and remyelination in the CNS. Exp. Neurol. 283, 501–511. doi: 10.1016/j.expneurol.2016.03.008, PMID: 26957369 PMC5010983

[ref32] Galloway D. A. Blandford S. N. Berry T. Williams J. B. Stefanelli M. Ploughman M.. (2019). miR-223 promotes regenerative myeloid cell phenotype and function in the demyelinated central nervous system. Glia 67, 857–869. doi: 10.1002/glia.23576, PMID: 30548333

[ref33] Galloway D. A. Carew S. J. Blandford S. N. Benoit R. Y. Fudge N. J. Berry T.. (2022). Investigating the NLRP3 inflammasome and its regulator miR-223-3p in multiple sclerosis and experimental demyelination. J. Neurochem. 163, 94–112. doi: 10.1111/jnc.15650 35633501

[ref34] Gebert L. F. R. MacRae I. J. (2019). Regulation of microRNA function in animals. Nat. Rev. Mol. Cell Biol. 20, 21–37. doi: 10.1038/s41580-018-0045-7, PMID: 30108335 PMC6546304

[ref35] Gholami S. Mirian M. Eftekhari S. M. Aliomrani M. (2020). Apamin administration impact on miR-219 and miR-155-3p expression in cuprizone induced multiple sclerosis model. Mol. Biol. Rep. 47, 9013–9019. doi: 10.1007/s11033-020-05959-6, PMID: 33174081

[ref36] Goldenberg M. M. (2012). Multiple sclerosis review. P T 37, 175–184. PMID: 22605909 PMC3351877

[ref37] Goldschmidt T. Antel J. König F. B. Brück W. Kuhlmann T. (2009). Remyelination capacity of the MS brain decreases with disease chronicity. Neurology 72, 1914–1921. doi: 10.1212/WNL.0b013e3181a8260a, PMID: 19487649

[ref38] Gruchot J. Weyers V. Göttle P. Förster M. Hartung H.-P. Küry P.. (2019). The molecular basis for remyelination failure in multiple sclerosis. Cells 8:825. doi: 10.3390/cells8080825, PMID: 31382620 PMC6721708

[ref39] Haghikia A. Haghikia A. Hellwig K. Baraniskin A. Holzmann A. Décard B. F.. (2012). Regulated microRNAs in the CSF of patients with multiple sclerosis: a case-control study. Neurology 79, 2166–2170. doi: 10.1212/WNL.0b013e3182759621, PMID: 23077021

[ref40] Hart A. D. Wyttenbach A. Perry V. H. Teeling J. L. (2012). Age related changes in microglial phenotype vary between CNS regions: grey versus white matter differences. Brain Behav. Immun. 26, 754–765. doi: 10.1016/j.bbi.2011.11.006, PMID: 22155499 PMC3381227

[ref41] Hemond C. C. Healy B. C. Tauhid S. Mazzola M. A. Quintana F. J. Gandhi R.. (2019). MRI phenotypes in MS: longitudinal changes and miRNA signatures. Neurol Neuroimmunol Neuroinflamm 6:e530. doi: 10.1212/NXI.0000000000000530, PMID: 30800720 PMC6384020

[ref42] Hiebert J. B. Shen Q. Thimmesch A. R. Pierce J. D. (2015). Traumatic brain injury and mitochondrial dysfunction. Am J Med Sci 350, 132–138. doi: 10.1097/MAJ.0000000000000506 26083647

[ref43] Huang J. K. Franklin R. J. M. (2012). “Chapter 12 - Current status of myelin replacement therapies in multiple sclerosis,” in Progress in brain research, functional neural transplantation III. eds. Dunnett S. B. Björklund A. (Elsevier), 219–231. 10.1016/B978-0-444-59544-7.00011-1 23186717

[ref44] Jia J. Jin H. Nan D. Yu W. Huang Y. (2021). New insights into targeting mitochondria in ischemic injury. Apoptosis 26, 163–183. doi: 10.1007/s10495-021-01661-5, PMID: 33751318

[ref45] Jolanda Münzel E. Williams A. (2013). Promoting remyelination in multiple sclerosis—recent advances. Drugs 73, 2017–2029. doi: 10.1007/s40265-013-0146-8, PMID: 24242317 PMC3853368

[ref46] Kamma E. Lasisi W. Libner C. Ng H. S. Plemel J. R. (2022). Central nervous system macrophages in progressive multiple sclerosis: relationship to neurodegeneration and therapeutics. J. Neuroinflammation 19:45. doi: 10.1186/s12974-022-02408-y, PMID: 35144628 PMC8830034

[ref47] Koch M. W. Zabad R. Giuliani F. Hader W. Lewkonia R. Metz L.. (2015). Hydroxychloroquine reduces microglial activity and attenuates experimental autoimmune encephalomyelitis. J. Neurol. Sci. 358, 131–137. doi: 10.1016/j.jns.2015.08.1525, PMID: 26344560

[ref48] Kremer D. Akkermann R. Küry P. Dutta R. (2019). Current advancements in promoting remyelination in multiple sclerosis. Mult. Scler. 25, 7–14. doi: 10.1177/1352458518800827, PMID: 30270725 PMC6389436

[ref49] Kremer D. Küry P. Dutta R. (2015). Promoting remyelination in multiple sclerosis: current drugs and future prospects. Mult. Scler. 21, 541–549. doi: 10.1177/1352458514566419, PMID: 25623245

[ref50] Kuhlmann T. Miron V. Cui Q. Wegner C. Antel J. Brück W. (2008). Differentiation block of oligodendroglial progenitor cells as a cause for remyelination failure in chronic multiple sclerosis. Brain 131, 1749–1758. doi: 10.1093/brain/awn096, PMID: 18515322

[ref51] Kuypers N. J. Bankston A. N. Howard R. M. Beare J. E. Whittemore S. R. (2016). Remyelinating oligodendrocyte precursor cell miRNAs from the Sfmbt2 cluster promote cell cycle arrest and differentiation. J. Neurosci. 36, 1698–1710. doi: 10.1523/JNEUROSCI.1240-15.2016, PMID: 26843650 PMC4737778

[ref52] Ladak A. A. Enam S. A. Ibrahim M. T. (2019). A review of the molecular mechanisms of traumatic brain injury. World Neurosurg. 131, 126–132. doi: 10.1016/j.wneu.2019.07.039 31301445

[ref53] Lassmann H. (2018). Multiple sclerosis pathology. Cold Spring Harb. Perspect. Med. 8:a028936. doi: 10.1101/cshperspect.a028936, PMID: 29358320 PMC5830904

[ref54] Lassmann H. Bradl M. (2017). Multiple sclerosis: experimental models and reality. Acta Neuropathol. 133, 223–244. doi: 10.1007/s00401-016-1631-4, PMID: 27766432 PMC5250666

[ref55] Lecca D. Marangon D. Coppolino G. T. Méndez A. M. Finardi A. Costa G. D.. (2016). MiR-125a-3p timely inhibits oligodendroglial maturation and is pathologically up-regulated in human multiple sclerosis. Sci. Rep. 6:34503. doi: 10.1038/srep34503, PMID: 27698367 PMC5048305

[ref56] Letzen B. S. Liu C. Thakor N. V. Gearhart J. D. All A. H. Kerr C. L. (2010). MicroRNA expression profiling of oligodendrocyte differentiation from human embryonic stem cells. PLoS One 5:e10480. doi: 10.1371/journal.pone.0010480, PMID: 20463920 PMC2864763

[ref57] Lewkowicz P. Cwiklińska H. Mycko M. P. Cichalewska M. Domowicz M. Lewkowicz N.. (2015). Dysregulated RNA-induced silencing complex (RISC) assembly within CNS corresponds with abnormal miRNA expression during autoimmune demyelination. J. Neurosci. 35, 7521–7537. doi: 10.1523/JNEUROSCI.4794-14.2015, PMID: 25972178 PMC6705439

[ref58] Li Y. Liu Z. Song Y. Pan J. Jiang Y. Shi X.. (2022). M2 microglia-derived extracellular vesicles promote white matter repair and functional recovery via miR-23a-5p after cerebral ischemia in mice. Theranostics 12, 3553–3573. doi: 10.7150/thno.68895, PMID: 35547763 PMC9065182

[ref59] Lin S.-T. Fu Y.-H. (2009). miR-23 regulation of lamin B1 is crucial for oligodendrocyte development and myelination. Dis. Model. Mech. 2, 178–188. doi: 10.1242/dmm.001065, PMID: 19259393 PMC2650193

[ref60] Lin S.-T. Huang Y. Zhang L. Heng M. Y. Ptácek L. J. Fu Y.-H. (2013). MicroRNA-23a promotes myelination in the central nervous system. Proc. Natl. Acad. Sci. U. S. A. 110, 17468–17473. doi: 10.1073/pnas.1317182110, PMID: 24101522 PMC3808585

[ref61] Lindner M. Heine S. Haastert K. Garde N. Fokuhl J. Linsmeier F.. (2008). Sequential myelin protein expression during remyelination reveals fast and efficient repair after central nervous system demyelination. Neuropathol. Appl. Neurobiol. 34, 105–114. doi: 10.1111/j.1365-2990.2007.00879.x, PMID: 17961136

[ref62] Liu X. S. Chopp M. Pan W. L. Wang X. L. Fan B. Y. Zhang Y.. (2017). MicroRNA-146a promotes oligodendrogenesis in stroke. Mol. Neurobiol. 54, 227–237. doi: 10.1007/s12035-015-9655-7, PMID: 26738853 PMC4935640

[ref63] Liu C. Li Y. Yu J. Feng L. Hou S. Liu Y.. (2013). Targeting the shift from M1 to M2 macrophages in experimental autoimmune encephalomyelitis mice treated with fasudil. PLoS One 8:e54841. doi: 10.1371/journal.pone.0054841, PMID: 23418431 PMC3572131

[ref64] Lopaisankrit T. Thammaroj J. (2023). Brain and spinal cord MRI findings in thai multiple sclerosis patients. J. Imaging 9:27. doi: 10.3390/jimaging9020027, PMID: 36826946 PMC9958745

[ref65] Loulier K. Ruat M. Traiffort E. (2006). Increase of proliferating oligodendroglial progenitors in the adult mouse brain upon Sonic hedgehog delivery in the lateral ventricle. J. Neurochem. 98, 530–542. doi: 10.1111/j.1471-4159.2006.03896.x 16805844

[ref66] Lucchinetti C. Brück W. Parisi J. Scheithauer B. Rodriguez M. Lassmann H. (1999). A quantitative analysis of oligodendrocytes in multiple sclerosis lesions. A study of 113 cases. Brain 122, 2279–2295. doi: 10.1093/brain/122.12.2279 10581222

[ref67] Marangon D. Boda E. Parolisi R. Negri C. Giorgi C. Montarolo F.. (2020). In vivo silencing of miR-125a-3p promotes myelin repair in models of white matter demyelination. Glia 68, 2001–2014. doi: 10.1002/glia.23819, PMID: 32163190

[ref68] Mason J. L. Ye P. Suzuki K. D’Ercole A. J. Matsushima G. K. (2000). Insulin-like growth Factor-1 inhibits mature oligodendrocyte apoptosis during primary demyelination. J. Neurosci. 20, 5703–5708. doi: 10.1523/JNEUROSCI.20-15-05703.2000, PMID: 10908609 PMC6772563

[ref69] Mazloumfard F. Mirian M. Eftekhari S.-M. Aliomrani M. (2020). Hydroxychloroquine effects on miR-155-3p and miR-219 expression changes in animal model of multiple sclerosis. Metab. Brain Dis. 35, 1299–1307. doi: 10.1007/s11011-020-00609-z, PMID: 32860610

[ref70] McCoy C. E. (2017). miR-155 dysregulation and therapeutic intervention in multiple sclerosis. Adv. Exp. Med. Biol. 1024, 111–131. doi: 10.1007/978-981-10-5987-2_5, PMID: 28921467

[ref71] Medina-Rodríguez E. M. Bribián A. Boyd A. Palomo V. Pastor J. Lagares A.. (2017). Promoting in vivo remyelination with small molecules: a neuroreparative pharmacological treatment for multiple sclerosis. Sci. Rep. 7:43545. doi: 10.1038/srep43545, PMID: 28256546 PMC5335257

[ref72] Meschkat M. Steyer A. M. Weil M.-T. Kusch K. Jahn O. Piepkorn L.. (2022). White matter integrity in mice requires continuous myelin synthesis at the inner tongue. Nat. Commun. 13:1163. doi: 10.1038/s41467-022-28720-y 35246535 PMC8897471

[ref73] Mi S. Miller R. H. Lee X. Scott M. L. Shulag-Morskaya S. Shao Z.. (2005). LINGO-1 negatively regulates myelination by oligodendrocytes. Nat. Neurosci. 8, 745–751. doi: 10.1038/nn1460 15895088

[ref74] Milbreta U. Lin J. Pinese C. Ong W. Chin J. S. Shirahama H.. (2019). Scaffold-mediated sustained, non-viral delivery of miR-219/miR-338 promotes CNS remyelination. Mol. Ther. 27, 411–423. doi: 10.1016/j.ymthe.2018.11.016, PMID: 30611662 PMC6369635

[ref75] Moody L. He H. Pan Y.-X. Chen H. (2017). Methods and novel technology for microRNA quantification in colorectal cancer screening. Clin. Epigenetics 9:119. doi: 10.1186/s13148-017-0420-9, PMID: 29090038 PMC5655825

[ref76] Morris J. K. Chomyk A. Song P. Parker N. Deckard S. Trapp B. D.. (2015). Decrease in levels of the evolutionarily conserved microRNA miR-124 affects oligodendrocyte numbers in zebrafish, *Danio rerio*. Invert. Neurosci. 15:4. doi: 10.1007/s10158-015-0180-1, PMID: 26159098

[ref77] Muñoz-San Martín M. Reverter G. Robles-Cedeño R. Buxò M. Ortega F. J. Gómez I.. (2019). Analysis of miRNA signatures in CSF identifies upregulation of miR-21 and miR-146a/b in patients with multiple sclerosis and active lesions. J. Neuroinflammation 16:220. doi: 10.1186/s12974-019-1590-5, PMID: 31727077 PMC6857276

[ref78] Neumann B. Baror R. van Wijngaarden P. Franklin R. J. (2017). Remyelination of regenerating axons. Acta Ophthalmol. 95. doi: 10.1111/j.1755-3768.2017.03525

[ref79] Nguyen L. H. Ong W. Wang K. Wang M. Nizetic D. Chew S. Y. (2019). Effects of miR-219/miR-338 on microglia and astrocyte behaviors and astrocyte-oligodendrocyte precursor cell interactions. Neural Regen. Res. 15, 739–747. doi: 10.4103/1673-5374.266922, PMID: 31638099 PMC6975139

[ref80] Nuzziello N. Vilardo L. Pelucchi P. Consiglio A. Liuni S. Trojano M.. (2018). Investigating the role of MicroRNA and transcription factor co-regulatory networks in multiple sclerosis pathogenesis. Int. J. Mol. Sci. 19:3652. doi: 10.3390/ijms19113652, PMID: 30463275 PMC6274935

[ref81] Osorio-Querejeta I. Carregal-Romero S. Ayerdi-Izquierdo A. Mäger I. Nash L. A. Wood M.. (2020). MiR-219a-5p enriched extracellular vesicles induce OPC differentiation and EAE improvement more efficiently than liposomes and polymeric nanoparticles. Pharmaceutics 12:186. doi: 10.3390/pharmaceutics12020186, PMID: 32098213 PMC7076664

[ref82] Otero-Ortega L. Laso-García F. Gómez-de Frutos M. D. C. Rodríguez-Frutos B. Pascual-Guerra J. Fuentes B.. (2017). White matter repair after extracellular vesicles administration in an experimental animal model of subcortical stroke. Sci. Rep. 7:44433. doi: 10.1038/srep44433, PMID: 28300134 PMC5353554

[ref83] Patrikios P. Stadelmann C. Kutzelnigg A. Rauschka H. Schmidbauer M. Laursen H.. (2006). Remyelination is extensive in a subset of multiple sclerosis patients. Brain 129, 3165–3172. doi: 10.1093/brain/awl217 16921173

[ref84] Pedersen B. K. Hoffman-Goetz L. (2000). Exercise and the immune system: regulation, integration, and adaptation. Physiol. Rev. 80, 1055–1081. doi: 10.1152/physrev.2000.80.3.1055, PMID: 10893431

[ref85] Pennati A. Nylen E. A. Duncan I. D. Galipeau J. (2020). Regulatory B cells normalize CNS myeloid cell content in a mouse model of multiple sclerosis and promote oligodendrogenesis and remyelination. J. Neurosci. 40, 5105–5115. doi: 10.1523/JNEUROSCI.2840-19.2020, PMID: 32430295 PMC7314404

[ref86] Piatek P. Namiecinska M. Domowicz M. Przygodzka P. Wieczorek M. Michlewska S.. (2019). MS CD49d+CD154+ lymphocytes reprogram oligodendrocytes into immune reactive cells affecting CNS regeneration. Cells 8:1508. doi: 10.3390/cells8121508, PMID: 31775315 PMC6953114

[ref87] Piatek P. Namiecinska M. Domowicz M. Wieczorek M. Michlewska S. Matysiak M.. (2020). Multiple sclerosis CD49d+CD154+ as myelin-specific lymphocytes induced during remyelination. Cells 9:15. doi: 10.3390/cells9010015, PMID: 31861635 PMC7017443

[ref88] Piket E. Zheleznyakova G. Y. Kular L. Jagodic M. (2019). Small non-coding RNAs as important players, biomarkers and therapeutic targets in multiple sclerosis: a comprehensive overview. J. Autoimmun. 101, 17–25. doi: 10.1016/j.jaut.2019.04.002, PMID: 31014917

[ref89] Pusic A. D. Kraig R. P. (2014). Youth and environmental enrichment generate serum exosomes containing miR-219 that promote CNS myelination. Glia 62, 284–299. doi: 10.1002/glia.22606, PMID: 24339157 PMC4096126

[ref90] Pusic K. M. Pusic A. D. Kraig R. P. (2016). Environmental enrichment stimulates immune cell secretion of exosomes that promote CNS myelination and may regulate inflammation. Cell. Mol. Neurobiol. 36, 313–325. doi: 10.1007/s10571-015-0269-4, PMID: 26993508 PMC4860060

[ref91] Qin C. Yang S. Chu Y.-H. Zhang H. Pang X.-W. Chen L.. (2022). Signaling pathways involved in ischemic stroke: molecular mechanisms and therapeutic interventions. Sig. Transduct. Target Ther. 7, 215–229. doi: 10.1038/s41392-022-01064-1, PMID: 35794095 PMC9259607

[ref92] Ransohoff R. M. (2012). Animal models of multiple sclerosis: the good, the bad and the bottom line. Nat. Neurosci. 15, 1074–1077. doi: 10.1038/nn.3168, PMID: 22837037 PMC7097342

[ref93] Ray S. K. Dixon C. E. Banik N. L. (2002). Molecular mechanisms in the pathogenesis of traumatic brain injury. Histol. Histopathol. 17, 1137–1152. doi: 10.14670/HH-17.1137 12371142

[ref94] Regev K. Healy B. C. Paul A. Diaz-Cruz C. Mazzola M. A. Raheja R.. (2018). Identification of MS-specific serum miRNAs in an international multicenter study. Neurol Neuroimmunol. Neuroinflamm. 5:e491. doi: 10.1212/NXI.0000000000000491, PMID: 30175165 PMC6117192

[ref95] Regev K. Paul A. Healy B. von Glenn F. Diaz-Cruz C. Gholipour T.. (2016). Comprehensive evaluation of serum microRNAs as biomarkers in multiple sclerosis. Neurol Neuroimmunol. Neuroinflamm. 3:e267. doi: 10.1212/NXI.0000000000000267, PMID: 27606352 PMC4996540

[ref96] Schmitz T. Chew L.-J. (2008). Cytokines and myelination in the central nervous system. ScientificWorldJournal 8, 1119–1147. doi: 10.1100/tsw.2008.140, PMID: 18979053 PMC2663591

[ref97] Selmaj I. Cichalewska M. Namiecinska M. Galazka G. Horzelski W. Selmaj K. W.. (2017). Global exosome transcriptome profiling reveals biomarkers for multiple sclerosis. Ann. Neurol. 81, 703–717. doi: 10.1002/ana.24931, PMID: 28411393

[ref98] Sheinerman K. S. Toledo J. B. Tsivinsky V. G. Irwin D. Grossman M. Weintraub D.. (2017). Circulating brain-enriched microRNAs as novel biomarkers for detection and differentiation of neurodegenerative diseases. Alzheimers Res. Ther. 9:89. doi: 10.1186/s13195-017-0316-0, PMID: 29121998 PMC5679501

[ref99] Shin D. Shin J.-Y. McManus M. T. Ptácek L. J. Fu Y.-H. (2009). Dicer ablation in oligodendrocytes provokes neuronal impairment in mice. Ann. Neurol. 66, 843–857. doi: 10.1002/ana.21927, PMID: 20035504 PMC2885004

[ref100] Song J. Li X. Li Y. Che J. Li X. Zhao X.. (2017). Biodegradable and biocompatible cationic polymer delivering microRNA-221/222 promotes nerve regeneration after sciatic nerve crush. Int. J. Nanomedicine 12, 4195–4208. doi: 10.2147/IJN.S132190, PMID: 28652727 PMC5473607

[ref101] Stolt C. C. Lommes P. Sock E. Chaboissier M.-C. Schedl A. Wegner M. (2003). The Sox9 transcription factor determines glial fate choice in the developing spinal cord. Genes Dev. 17, 1677–1689. doi: 10.1101/gad.259003, PMID: 12842915 PMC196138

[ref102] Stolt C. C. Rehberg S. Ader M. Lommes P. Riethmacher D. Schachner M.. (2002). Terminal differentiation of myelin-forming oligodendrocytes depends on the transcription factor Sox10. Genes Dev. 16, 165–170. doi: 10.1101/gad.215802 11799060 PMC155320

[ref103] Stolt C. C. Schlierf A. Lommes P. Hillgärtner S. Werner T. Kosian T.. (2006). SoxD proteins influence multiple stages of oligodendrocyte development and modulate SoxE protein function. Dev. Cell 11, 697–709. doi: 10.1016/j.devcel.2006.08.011, PMID: 17084361

[ref104] Swanson K. V. Deng M. Ting J. P.-Y. (2019). The NLRP3 inflammasome: molecular activation and regulation to therapeutics. Nat. Rev. Immunol. 19, 477–489. doi: 10.1038/s41577-019-0165-0, PMID: 31036962 PMC7807242

[ref105] Tepavčević V. Lubetzki C. (2022). Oligodendrocyte progenitor cell recruitment and remyelination in multiple sclerosis: the more, the merrier? Brain 145, 4178–4192. doi: 10.1093/brain/awac307, PMID: 36093726

[ref106] Tripathi A. Volsko C. Garcia J. P. Agirre E. Allan K. C. Tesar P. J.. (2019). Oligodendrocyte intrinsic miR-27a controls myelination and remyelination. Cell Rep. 29, 904–919.e9. doi: 10.1016/j.celrep.2019.09.020, PMID: 31644912 PMC6874400

[ref107] Uchida N. Chen K. Dohse M. Hansen K. D. Dean J. Buser J. R.. (2012). Human neural stem cells induce functional myelination in mice with severe dysmyelination. Sci. Transl. Med. 4:155ra136. doi: 10.1126/scitranslmed.3004371, PMID: 23052293 PMC3864816

[ref108] Uyeda A. Muramatsu R. (2020). Molecular mechanisms of central nervous system axonal regeneration and remyelination: a review. Int. J. Mol. Sci. 21:8116. doi: 10.3390/ijms21218116, PMID: 33143194 PMC7662268

[ref109] Valério-Gomes B. Guimarães D. M. Szczupak D. Lent R. (2018). The absolute number of oligodendrocytes in the adult mouse brain. Front. Neuroanat. 12:90. doi: 10.3389/fnana.2018.00090, PMID: 30425626 PMC6218541

[ref110] van Langelaar J. Rijvers L. Smolders J. van Luijn M. M. (2020). B and T cells driving multiple sclerosis: identity, mechanisms and potential triggers. Front. Immunol. 11:760. doi: 10.3389/fimmu.2020.00760 32457742 PMC7225320

[ref111] Vistbakka J. Sumelahti M.-L. Lehtimäki T. Elovaara I. Hagman S. (2018). Evaluation of serum miR-191-5p, miR-24-3p, miR-128-3p, and miR-376c-3 in multiple sclerosis patients. Acta Neurol. Scand. 138, 130–136. doi: 10.1111/ane.12921 29527713

[ref112] Wang C.-Y. Deneen B. Tzeng S.-F. (2017). MicroRNA-212 inhibits oligodendrocytes during maturation by down-regulation of differentiation-associated gene expression. J. Neurochem. 143, 112–125. doi: 10.1111/jnc.14138, PMID: 28771716

[ref113] Wang H. Moyano A. L. Ma Z. Deng Y. Lin Y. Zhao C.. (2017). miR-219 cooperates with miR-338 in myelination and promotes myelin repair in the CNS. Dev. Cell 40, 566–582.e5. doi: 10.1016/j.devcel.2017.03.001, PMID: 28350989 PMC5569304

[ref114] Wang S. Sdrulla A. D. diSibio G. Bush G. Nofziger D. Hicks C.. (1998). Notch receptor activation inhibits oligodendrocyte differentiation. Neuron 21, 63–75. doi: 10.1016/s0896-6273(00)80515-2 9697852

[ref115] Wiggermann V. Endmayr V. Hernández-Torres E. Höftberger R. Kasprian G. Hametner S.. (2023). Quantitative magnetic resonance imaging reflects different levels of histologically determined myelin densities in multiple sclerosis, including remyelination in inactive multiple sclerosis lesions. Brain Pathol.:e13150. doi: 10.1111/bpa.13150, PMID: 36720269 PMC10580011

[ref116] Wittstatt J. Weider M. Wegner M. Reiprich S. (2020). MicroRNA miR-204 regulates proliferation and differentiation of oligodendroglia in culture. Glia 68, 2015–2027. doi: 10.1002/glia.23821, PMID: 32176386

[ref117] Wlodarczyk A. Holtman I. R. Krueger M. Yogev N. Bruttger J. Khorooshi R.. (2017). A novel microglial subset plays a key role in myelinogenesis in developing brain. EMBO J. 36, 3292–3308. doi: 10.15252/embj.201696056, PMID: 28963396 PMC5686552

[ref118] Woodruff R. H. Fruttiger M. Richardson W. D. Franklin R. J. M. (2004). Platelet-derived growth factor regulates oligodendrocyte progenitor numbers in adult CNS and their response following CNS demyelination. Mol. Cell. Neurosci. 25, 252–262. doi: 10.1016/j.mcn.2003.10.014, PMID: 15019942

[ref119] Xiao Y. Geng F. Wang G. Li X. Zhu J. Zhu W. (2019). Bone marrow-derived mesenchymal stem cells-derived exosomes prevent oligodendrocyte apoptosis through exosomal miR-134 by targeting caspase-8. J. Cell. Biochem. 120, 2109–2118. doi: 10.1002/jcb.27519, PMID: 30191592

[ref120] Zhao X. He X. Han X. Yu Y. Ye F. Chen Y.. (2010). MicroRNA-mediated control of oligodendrocyte differentiation. Neuron 65, 612–626. doi: 10.1016/j.neuron.2010.02.018, PMID: 20223198 PMC2855245

[ref121] Zheng X. Huang H. Liu J. Li M. Liu M. Luo T. (2018). Propofol attenuates inflammatory response in LPS-activated microglia by regulating the miR-155/SOCS1 pathway. Inflammation 41, 11–19. doi: 10.1007/s10753-017-0658-6, PMID: 28875362

[ref122] Zurawska A. Mycko M. P. Selmaj K. W. (2019). Circular RNAs as a novel layer of regulatory mechanism in multiple sclerosis. J. Neuroimmunol. 334:576971. doi: 10.1016/j.jneuroim.2019.576971, PMID: 31163273

